# Isolation and identification of pathogens of *Morchella sextelata* bacterial disease

**DOI:** 10.3389/fmicb.2023.1231353

**Published:** 2023-11-06

**Authors:** Xuetai Zhu, Kaili Ma, Mingyue Sun, Jinming Zhang, Lijuan Liu, Shiquan Niu

**Affiliations:** College of Life Sciences, Northwest Normal University, Lanzhou, China

**Keywords:** *Morchella sextelata*, bacterial disease, isolation and identification, biological property, pathogenic bacteria

## Abstract

Morel mushroom (*Morchella* spp.) is a rare edible and medicinal fungus distributed worldwide. It is highly desired by the majority of consumers. Bacterial diseases have been commonly observed during artificial cultivation of *Morchella sextelata*. Bacterial pathogens spread rapidly and cause a wide range of infections, severely affecting the yield and quality of *M. sextelata*. In this study, two strains of bacterial pathogens, named M-B and M-5, were isolated, cultured, and purified from the tissues of the infected *M. sextelata*. Koch’s postulates were used to determine the pathogenicity of bacteria affecting *M. sextelata*, and the pathogens were identified through morphological observation, physiological and biochemical analyses, and 16S rRNA gene sequence analysis. Subsequently, the effect of temperature on the growth of pathogenic bacteria, the inhibitory effect of the bacteria on *M. sextelata* on plates, and the changes in mycelial morphology of *M. sextelata* mycelium were analyzed when *M. sextelata* mycelium was double-cultured with pathogenic bacteria on plates. The results revealed that M-B was *Pseudomonas chlororaphis* subsp. *aureofaciens* and M-5 was *Bacillus subtilis*. Strain M-B started to multiply at 10–15°C, and strain M-5 started at 15–20°C. On the plates, the pathogenic bacteria also produced significant inhibition of *M. sextelata* mycelium, and the observation of mycelial morphology under the scanning electron microscopy revealed that the inhibited mycelium underwent obvious drying and crumpling, and the healthy mycelium were more plump. Thus, this study clarified the pathogens, optimal growth environment, and characteristics of *M. sextelata* bacterial diseases, thereby providing valuable basic data for the disease prevention and control of *Morchella* production.

## 1. Introduction

Morels (*Morchella* spp.), a valuable class of fungi used for food and medicine, is the collective name of the fungi belong to the *Morchella* genus (Zhao, 2018; Du et al., 2020). Because of its uneven cap, the shape of this fungus resembles the belly of a sheep and hence named accordingly (Du et al., 2019; Sun et al., 2019). The fungus is rich in amino acids, adenosine, and other flavorful substances. Because of their crisp texture and unique flavor, these delicious mushrooms often serve as a perfect addition to any meal (Rotzoll et al., 2006; Wang et al., 2016). Morels are also rich in protein, lipids, fiber, vitamins, minerals, and other nutrients (Vieira et al., 2016; Tietel and Masaphy, 2018), and polysaccharides isolated from morels have good biological activities such as antioxidant, anti-inflammatory, antitumor, immunomodulatory, and regulation of intestinal flora (Chang and Wasser, 2012; Vieira Gomes et al., 2019; Goldman et al., 2021; Xu et al., 2022; Li et al., 2023). Other chemical constituents of morels such as phenolics, ascorbic acid, and carotenoids have attracted research interest because of their excellent biological activity, mainly its high antioxidant activity (Soobrattee et al., 2005; Leal et al., 2013; Tietel and Masaphy, 2018; Vieira Gomes et al., 2019). In traditional medicine, morels have been used as an antiseptic. The powder of the fruiting bodies is dumped into the wound to facilitate the rapid healing of wounds. They are also used to treat stomach pain (Mahmood et al., 2011).

Because of its high economic value, indoor cultivation of morel mushrooms has received considerable attention worldwide (Du et al., 2015). Outdoor cultivation of morel mushrooms was first performed in France and was reported by Roze (1882) in relation to *Jerusalem artichokes* (Liu et al., 2018). Successful indoor cultivation of morels was first reported in 1982 by Owwer (1982). This subsequently resulted in three successive patents for morel mushroom cultivation (US Patents 4594809, 4757640, and 4866878) (Du et al., 2012; Liu et al., 2018). However, this indoor morel cultivation method could not be applied on a large scale due to inconsistent harvesting yields. Based on the utilization of exogenous nutrients and successful domestication of easy-to-cultivate varieties since 2012, 16,466 ha of outdoor cultivation of morels has been achieved in mainland China during the 2021–2022 season (Tan et al., 2019; Shi et al., 2022). As morel cultivation is rapidly expanding, diseases are increasingly becoming the greatest threat to morel mushroom production (Tan et al., 2021a). Diseases affecting morel mushroom production are mainly caused by bacteria and fungi. More current studies have focused on fungal diseases, such as stipe rot disease caused by the *Fusarium incarnatum*–*F. equiseti* species complex (Guo et al., 2016) and by *Purpureocillium lilacinum* (Masaphy, 2022), cobweb disease caused by *Hypomyces*/*Cladobotryum* species (Lan et al., 2020), white mold disease caused by *Paecilomyces penicillatus* (He et al., 2018; Yu et al., 2022), and pileus rot disease caused by *Diploöspora longispora* (He et al., 2018; Liu et al., 2018; Sun et al., 2023). However, studies on bacterial diseases of morels are relatively scarce. At least two types of bacterial diseases were initially identified, namely soft rot and red-stipe diseases (Liu et al., 2019), but their specific causative pathogens have not yet been identified. Recent studied reported red-stipe disease in *Morchella sextelata* and identified several important genes, metabolites, and pathways through transcriptomic and metabolomic analyses of the infected *M. sextelata*. Based on the results, the possibility of *F. nematophilum* being the causative pathogen of red-stipe disease was ruled out (Yang et al., 2023).

In April 2022, a disease in the main area of morel production in the southern part of Gansu Province, China, severely affected *M. sextelata* production. It manifested as symptoms of red-stipe disease, stopping the growth of fruiting bodies. During the disease outbreak, the whole stipe was reddish brown, and then, the color gradually deepened and spread from the bottom. The whole fruit body turned red and released a foul odor. With the disease course, *Morchella* lost its vitality, stopped growing, and eventually rot. The disease can lead to a major reduction in yield or even extinction in a short period, thereby exerting a great impact on morel mushroom cultivation. In this study, field survey, sample collection, isolation, identification, and rewiring of suspected pathogenic bacteria were performed for the widespread occurrence of *M. sextelata* red-stipe disease in the main *Morchella* production area in the southern part of Gansu Province. The pathogenic strain causing red-stipe disease in *M. sextelata* was identified on the basis of Koch’s postulates as well as and biological characterization and 16S rRNA gene sequence analysis. The study thus provides basic data for the accurate identification of the causative agent and prevention and control of the bacterial disease affecting *M. sextelata* in the field.

## 2. Materials and methods

### 2.1. Collection of samples of infected *M. sextelata* and isolation of pathogenic bacteria

The infected *M. sextelata* fruiting bodies were collected from the *Morchella* cultivation base in Chengxian, Longnan City, Gansu Province. In total, 30 samples were collected, 20 diseased samples, and 10 non-diseased samples, packed in clean self-sealing bags, and stored in a 4°C refrigerator at the laboratory for pathogen isolation. The samples were first rinsed with tap water to remove the soil attached and then rinsed with sterile water. The samples were first immersed in 75% ethanol for approximately 10 s and then in 0.1% mercuric chloride for 10 s, and washed 3 times with sterile water. Then, the materials were cut into 1–2-mm tissue slices with sterile blades and inoculated on Luria–Bertani (LB) solid medium. The LB plates with the slices were incubated in a thermostatic electric incubator at 30°C for 24 h. The bacteria were isolated from the LB agar plates using the scratch method. Single colonies with different morphologies were picked for isolation and purification. A single colony was picked and incubated on the LB plate again by streaking for 24 h until a single colony was obtained. A part of the purified strain was used for subsequent experiments, and the remaining part was stored in 20% glycerol at −80°C for future use.

### 2.2. Pathogenicity test

Pathogenicity tests were conducted according to Koch’s postulates. *Morchella sextelata* was selected as the main cultivar of *Morchella*. The pathogenic strains to be tested were inoculated in LB liquid medium for activation and incubated at 30°C with 160 r/min oscillation. Overnight bacterial cultures (1 × 10^8^ CFU/mL) grown in LB medium were injected into the ascocarps of morels by using a 1-mL sterile syringe. The experiment consisted of three treatment groups and three control groups. The first treatment group was only inoculated with M-B bacterial cultures, the second treatment group was only inoculated with M-5 bacterial cultures, the third treatment group was inoculated with both M-B and M-5 bacterial cultures, the fourth group was inoculated with sterilized M-B cultures as a killed bacterial control; the fifth group was inoculated with sterilized M-5 cultures as a killed bacterial control. The LB medium treatment was used as the blank control. Three replicates were established for each treatment group. In order to simulate field cultivation conditions in the laboratory, the inoculated *M. sextelata* was cultivated in a constant temperature incubation room at 17°C and 85% humidity. The disease incidence rate and symptoms were recorded. Two days after inoculation, some symptoms were observed on the surface. The pathogenic bacteria were reisolated from the infected *M. sextelata* fruiting bodies on LB plates, and the characteristics of the reisolated bacteria were compared with those of the original culture.

### 2.3. Identification of pathogens

The purified pathogenic bacteria were inoculated on LB plates and incubated at 30°C for 24 h. After observing the growth and morphological characteristics of the colonies, individual colonies were picked for microscopic examination. The isolates were stained using the Gram staining kit. The physiological and biochemical indicators of the isolated pathogenic bacteria isolated were determined by referring to the bacterial identification method presented in the Manual of Identification of Common Bacterial Systems (Dong and Cai, 2001). The physiological indicators included colony morphology, growth temperature, pseudocyanine production, fluorochrome production, utilization of carbon sources (glucose, mannitol, sucrose, trehalose, and fructose), and utilization of nitrogen sources (yeast powder, beef paste, ammonium sulfate, peptone, and sodium nitrate). The biochemical tests conducted included gelatin liquefaction, starch hydrolysis, H_2_S production, Voges–Proskauer test, methyl red test, and catalase and urease determination.

All strains isolated were subjected to phylogenetic analysis. DNA was extracted using the bacterial DNA extraction kit. The 16S rRNA gene fragment of the strain was amplified through PCR by using bacterial universal primers 27F (5′-AGAGTTTGATCATGGCTCAG-3′) and 1492R (5′-GGYTACCTTGTTACGACTT-3′). PCR reaction conditions were pre-denaturation at 95°C for 5 min, denaturation at 95°C for 1 min, annealing at 55°C for 30 s, extension at 72°C for 1 min, 34 cycles, extension at 72°C for 5 min amplification (Dou et al., 2018). Then, 1% agarose gel electrophoresis was performed to detect the PCR products. The PCR products were then selected and sent to Jinweizhi Biotechnology Co., Ltd., for sequencing. The sequences were compared with the known sequences in the NCBI database by using BLAST. The phylogenetic tree was constructed using the overlay method (neighbor joining) with MEGA 11.0 software and subjected to the bootstrap test (1,000). The outgroups were *Azotobacter chroococcum* and *Aeribacillus pallidus*, respectively.

Bacterial morphology was observed under scanning electron microscopy (SEM). The actively growing bacterial solution was centrifuged at 8,000 rpm for 3–5 min, and the supernatant was discarded and poured into 2.5% glutaraldehyde and fixed overnight. The samples were then washed 3 times with phosphate buffered saline (PBS), fixed in 1% osmium solution for 4–6 h, washed 3 times with phosphate buffered saline, then dehydrated once in 30, 50, 70, 85, and 95% ethanol, and treated twice with 100% ethanol for 15–20 min each. The samples are then subjected to critical-point drying and gold spraying before being ready for SEM (ULTRA Plus-type, Zeiss Company, Germany) (Xie et al., 2005).

### 2.4. Inhibition of growth and changes in mycelial morphology of *M. sextelata* by pathogenic bacteria on plates

The isolated pathogenic bacteria were inoculated on LB plates and incubated at 30°C for 24 h for activation. The laboratory-preserved *M. sextelata* strain ZW111 was inoculated on potato dextrose agar (PDA) plates and incubated at 20°C for 4 days for activation. The plate standoff method (Wang W. X. et al., 2014) was used to detect the inhibitory effect of the pathogenic bacteria. *M. sextelata* ZW111’s cake was placed in the center of the PDA plate. The antagonistic bacterial cake (diameter = 8 mm) was placed on three points at equal distance (2.5 cm) from the center of the *M. sextelata*. Three parallels were run for each treatment. The control group plate only contained *M. sextelata* ZW111. Two other *B. subtilis* preserved in this laboratory were selected for testing to preliminarily investigate whether all *B. subtilis* species inhibited *M. sextelata* growth or only the strain M-5, isolated in this study, inhibited *M. sextelata*. *P. chlororaphis* subsp. *aureofaciens* exhibits a strong antifungal activity (Raio et al., 2011; Zhang et al., 2021), and so, other strains were not tested. All plates were incubated at 25°C for 4 days in a constant temperature incubator. The pathogenic bacteria in the plates were observed for their inhibitory effect on *M. sextelata*, and observed morphological changes in the mycelium under SEM. The samples were taken from the mycelium of *M. sextelata* located in the marginal region of the inhibition zone, and the subsequent fixation, dehydration, drying and spraying gold treatments of the samples were referred to in 2.3.

### 2.5. Effect of temperature on the growth of pathogenic bacteria

The occurrence of bacterial diseases of *M. sextelata* during cultivation is closely related to the influence of temperature, especially high temperature and high humidity, we determined the effect of temperature on the growth of pathogenic bacteria to provide a better theoretical reference for preventing and controlling field diseases of morels. The prepared LB liquid medium was divided into test tubes, with each tube containing 10 mL LB medium. Then, 0.1 mL of bacterial solution (1 × 10^8^ CFU/mL) was added to each of the test tubes. The inoculated tubes were placed on a shaker at 4, 10, 15, 20, 25, 28, 30, 35, 37, 40, and 42°C, 160 r/min and incubated with shaking for 24 h. Three replicates were established set for each group of temperature, and the culture medium without any inoculum was used as the control. After 24 h of incubation, the OD_600*nm*_ values of the bacterial broth incubated at different temperatures were measured using a ultraviolet-visible spectrophotometer and recorded (Zhou, 2013).

### 2.6. Identification of cellulase production by pathogenic strains and determination of enzyme activity

The isolated pathogenic bacteria were inoculated in carboxymethyl cellulose (CMC) agar medium (0.5% CMC-Na, 0.1% NaNO_3_, 0.1% K_2_HPO_4_, 0.1% NaCl, 0.05% MgSO_4_, 0.05% yeast extract, 1.5% agar) and incubated at a constant temperature of 37°C. After incubation, the CMC agar plates were flooded with 0.2% Congo red for 30 min, and destained by washing twice with 1 mol/L NaCl solution for 20 min. Standard cellulase solution (MAKLIN Reagent Co.) was used as a positive control and inactivated enzyme solution as a negative control. The activity zones had observed against a red background after Congo red staining (Zhang et al., 2022).

The filter paper enzyme activity (FPase) of cellulase was measured through the amount of reducing sugars liberated during hydrolysis using filter paper as substrate by the DNS (3,5-dinitrosalicylic acid) method (Ghose, 1987; Xie et al., 2023). The strains to be tested were inoculated into cellulase production broth (1% CMC-Na, 1% peptone, 0.00075% FeSO_4_⋅7H_2_O, 0.00025% MnSO_4_⋅H_2_O, 0.0002% ZnSO_4_), incubated at 30°C for 3–4 days at 160 r/min, and centrifuged at 6,000 r/min for 10 min, and the supernatant was the crude enzyme solution. Cut the filter paper into 1 cm × 3 cm strips, roll them into small rolls and place them in a 5 mL test tube, add 1.75 mL of acetic acid-sodium acetate buffer (pH = 4.8) and 0.25 mL of enzyme solution, respectively. Place the tube in a water bath at 50°C for 1 h, then the reaction was terminated by 1 mL DNS. The color of the mixture was developed by incubating it for 10 min at 100°C. The inactivated crude enzyme solution was treated as above and used as a control. The absorbance was measured at 540 nm by using an ultraviolet-visible spectrophotometer. A standard curve was plotted using glucose. The equation of the standard curve is *y* = 0.5515 × +0.0206 and the correlation coefficient is *R*^2^ = 0.9996 (Supplementary Table 3). One unit (U) is defined as the amount of enzyme required to produce 1 μmol of glucose equivalent released per minute under the conditions described above (Li et al., 2020; Sarangthem et al., 2023).

### 2.7. Identification of chitinase production by pathogenic strains and determination of enzyme activity

The isolated pathogenic bacteria were inoculated on colloidal chitin agar medium [1% colloidal chitin, 0.5% NaCl, 0.05% MgSO_4_⋅7H_2_O, 0.07% K_2_HPO_4_, 0.03% KH_2_PO_4_, 1% (NH_4_)_2_SO_4_, 0.3% yeast extract powder, and 1.5% agar] and incubated at a constant temperature of 37°C. Colloidal chitin was prepared according to the method by Sandhya et al. (2004). After incubation, the plates were stained with 0.2% Congo red using the same method as in 2.6. Standard chitinase solution (MAKLIN Reagent Co.) was used as a positive control and inactivated enzyme solution as a negative control (Linda et al., 2018).

The chitinase activity of the strains was also determined by the DNS (3,5-dinitrosalicylic acid) method as in 2.6. The strains were inoculated into colloidal chitin broth (1% colloidal chitin, 0.05% MgSO_4_⋅7H_2_O, 1.2% urea, 0.03% KH_2_PO_4_, 0.5% NaCl, 0.07% K_2_HPO_4_, 0.3% yeast extract powder), the supernatant was the crude enzyme solution. Mix 0.5 ml crude enzyme solution with 0.5 ml colloid chitin and 0.5 ml PBS by volume. The reaction was carried out in a water bath at 37°C for 30 min, then the reaction was terminated by 1 mL DNS. The absorbance was measured at 540 nm. The inactivated crude enzyme solution was treated as above and used as a control. A standard curve was plotted using N-acetyl-D-glucosamine. The equation of the standard curve is *y* = 0.4063 × −0.0384 and the correlation coefficient is *R*^2^ = 0.9994 (Supplementary Table 4). One unit (U) is defined as the amount of enzyme required to catalyze the production of 1 μmol of N-acetyl-D-glucosamine from the substrate in 1 min under suitable conditions as one enzyme activity unit (U) (Du et al., 2021).

### 2.8. Statistical analysis

All experiments were repeated in triplicate. The data were processed using the SPASS statistical software to perform an ANOVA [least-significant difference (LSD) test and Duncan’s multiple range test, *p* < 0.05]. Origin software was used for statistical analysis and graph drawing.

## 3. Results

### 3.1. Collection of samples and culture properties of isolates from infected *M. sextelata*

**FIGURE 1:**
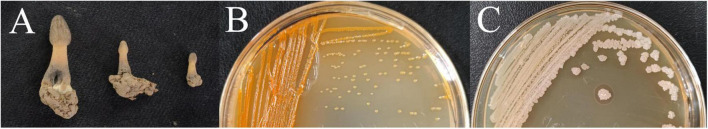
Samples of infected *M. sextelata* collected **(A)**; morphological characteristics of isolate colonies M-B **(B)** and M-5 **(C)**.

The collected infected *M. sextelata* samples were reddish in color and had a foul odor (Figure 1A). We isolated two types of bacteria, named M-B and M-5, from the diseased *M. sextelata*, and we did not isolate these bacteria from the undiseased *M. sextelata*. Strain M-B was inoculated on LB solid medium and incubated at 30°C for 24 h. The colonies appeared orange, smooth, and opaque with a round, slightly elevated surface (Figure 1B). Strain M-5 was inoculated on LB solid medium and incubated at 30°C for 24 h. After incubation, the colonies appeared creamy white, subcircular, with irregular edges, rough and opaque, and a dry, wrinkled surface (Figure 1C).

### 3.2. Pathogenicity testing

**FIGURE 2:**
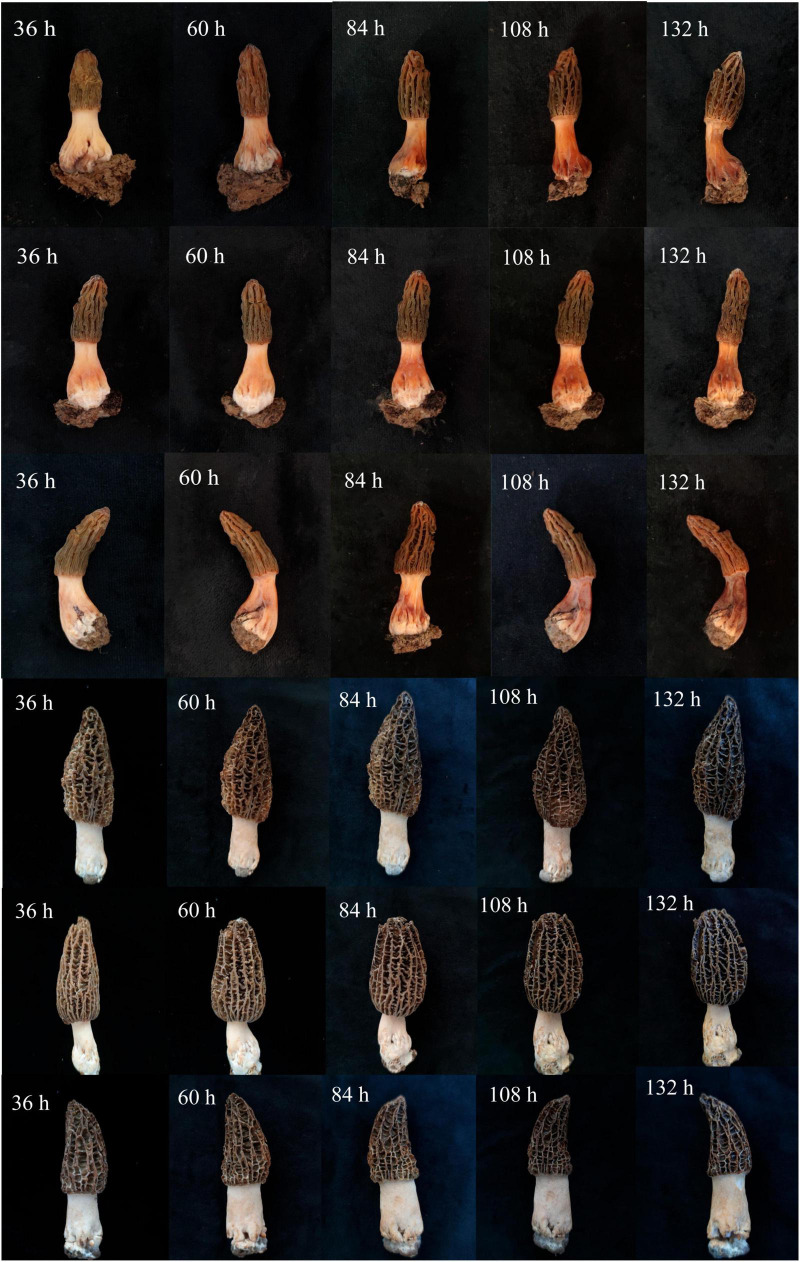
Pathogenicity test of isolates. The first row of *M. sextelata* was inoculated with only M-5 ramets, the second row of *M. sextelata* was inoculated with M-B alone, and the third row of *M. sextelata* was inoculated with both M-5 and M-B, the fourth row of control group was inoculated with sterilized M-B (CK1), the fifth row of control group was inoculated with sterilized M-5 (CK2), and the sixth row of control group was inoculated with LB blank medium (CK3).

The treated *M. sextelata* were placed in the incubation room at 17°C and 85% humidity. At 36 h after inoculation, reddish spots started to appear from the base of the stipe of *M. sextelata* in all bacterial solution treatment groups (Figure 2). After 60 h, the whole stipe began to turn red, and the red color of the stipe was the deepest in the mixed treatment group, and the M-5-treated *M. sextelata* started to produce a foul odor (Figure 2). At 84 h, the whole mushroom body in all bacterial solution treatment groups began to turn red and the red color continued to deepen (Figure 2). A total of 108 h later, the mushroom body began to turn reddish brown and soft rot (Figure 2). At 132 h, the whole body was dark red, wilted, and crumpled, accompanied by a large amount of unpleasant odor (Figure 2). Similar to field symptoms. The three control groups inoculated with sterilized M-B bacterial solution, sterilized M-5 bacterial solution, and LB blank medium showed slight browning over time, with no softening of the fruiting body and without symptoms of disease. Pathogenic bacteria were reisolated from these infected *M. sextelata*. These isolates exhibited similar morphologies on LB solid plates, satisfying Koch’s postulates.

### 3.3. Identification of pathogens

According to the results of Gram staining, strain M-5 (Supplementary Figure 1A) was stained purple and exhibited a rod-shaped body, with bluntly rounded ends and budding spores. It was tentatively determined to be Gram-positive. Morphological analysis through SEM (Supplementary Figure 1B) revealed that the bacterium was rod-shaped with no pods and a size of (0.6–0.7) μm × (2.3–1.4) μm. Its endospore was located in the center of the body or slightly deviated and did not expand after the bacterial cell became oval. Strain M-B (Supplementary Figure 1C) was stained red and exhibited a rod-shaped body. It was tentatively determined to be Gram-negative. SEM (Supplementary Figure 1D) revealed that the bacterium was rod-shaped, with a size of (0.4–0.5) μm × (1–1.3) μm.

Physiological and biochemical characterization of strains M-B and M-5 showed (Table 1) that both strains M-B and M-5 were able to utilize glucose, mannitol, sucrose, trehalose, fructose, yeast, beef extract, ammonium sulfate, peptone, sodium nitrate. Strain M-B was negative for starch hydrolysis, H_2_S production, methyl red test, positive for gelatin liquefaction, Voges–Proskauer test, catalase and urease reaction. Strain M-5 was negative for H_2_S production, methyl red test and urease reaction, and positive for gelatin liquefaction, catalase, Voges–Proskauer test and urease reaction. According to the physiological and biochemical tests, and combined with morphological characteristics, according to the Manual of Identification of Common Bacterial Systems, the strain M-B was preliminarily identified as *Pseudomonas chlororaphis* subsp. *aureofaciens* and strain M-5 was preliminarily identified as *Bacillus subtilis*.

**TABLE 1:** Physiological and biochemical identification of isolated strains.

Characteristics	M-B	M-5
**Shape**	**Rods**	**Rods**
Size (μm)	(0.4–0.5) × (1–1.3)	(0.6–0.7) × (2.3–1.4)
Gram’s reaction	−	+
Pyocyanin production	−	−
Fluorescent pigment	+	−
Glucose	+	+
Mannitol	+	+
Sucrose	+	+
Trehalose	+	+
Fructose	+	+
Yeast	+	+
Beef extract	+	+
Ammonium sulfate	+	+
Peptone	+	+
Sodium nitrate	+	+
Gelatin liquefaction	+	+
Starch hydrolysis	−	+
H_2_S formation	−	−
Voges–Proskauer test	+	+
Methyl red test	−	−
Catalase	+	+
Urease	+	+

+, positive effect;−, no effect.

**FIGURE 3:**
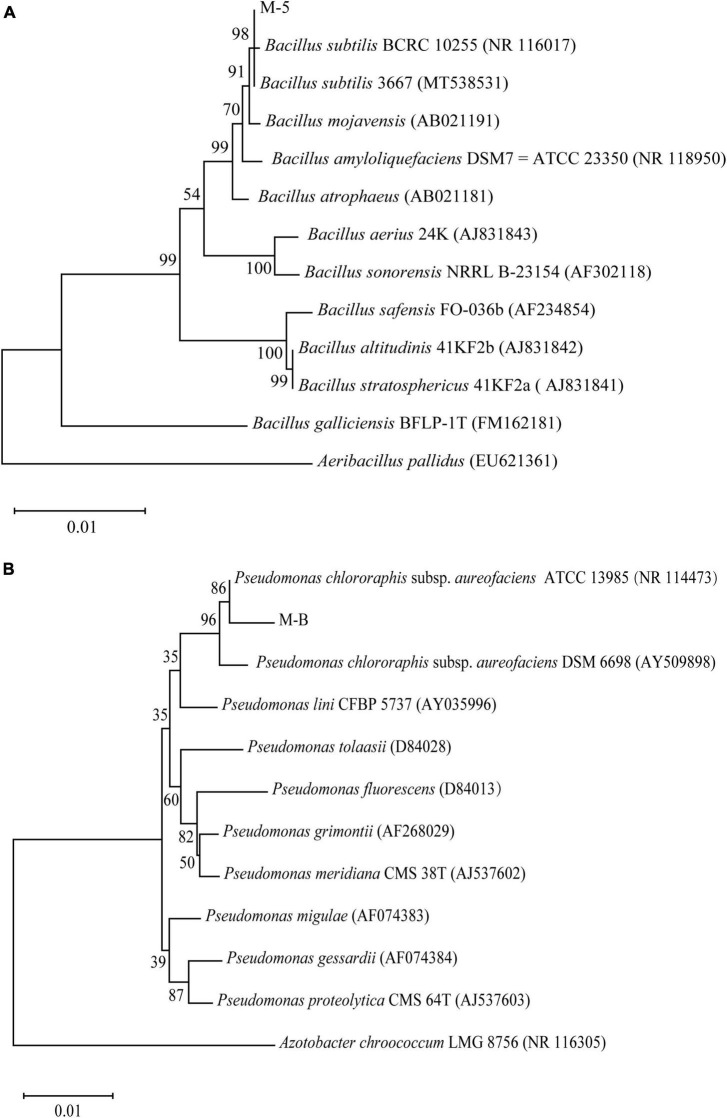
Phylogenetic analysis based on sequencing of the 16S ribosomal RNA gene. Bootstrap values (%) presented at the branches were calculated from 1,000 replications. Strain M-5 **(A)**; strain M-B **(B)**.

After sequencing was completed, the sequences were compared with the known sequences in the NCBI database by using BLAST. The sequences with a high homology were downloaded and compared using MEGA 11.0 software to construct a phylogenetic tree by using the neighbor-joining method. The results revealed (Figure 3) that the isolated strains belonged to two main groups. M-5 and *B. subtilis* converged in the same species, and so, the bacterium was tentatively identified as *B. subtilis*. M-B and *P. chlororaphis* subsp. *aureofaciens* converged in the same species, and therefore, the bacterium was tentatively identified as *P. chlororaphis* subsp. *aureofaciens*.

### 3.4. Inhibitory effect of pathogenic bacteria on *M. sextelata* on plates and changes in mycelial morphology of *M. sextelata*

**FIGURE 4:**
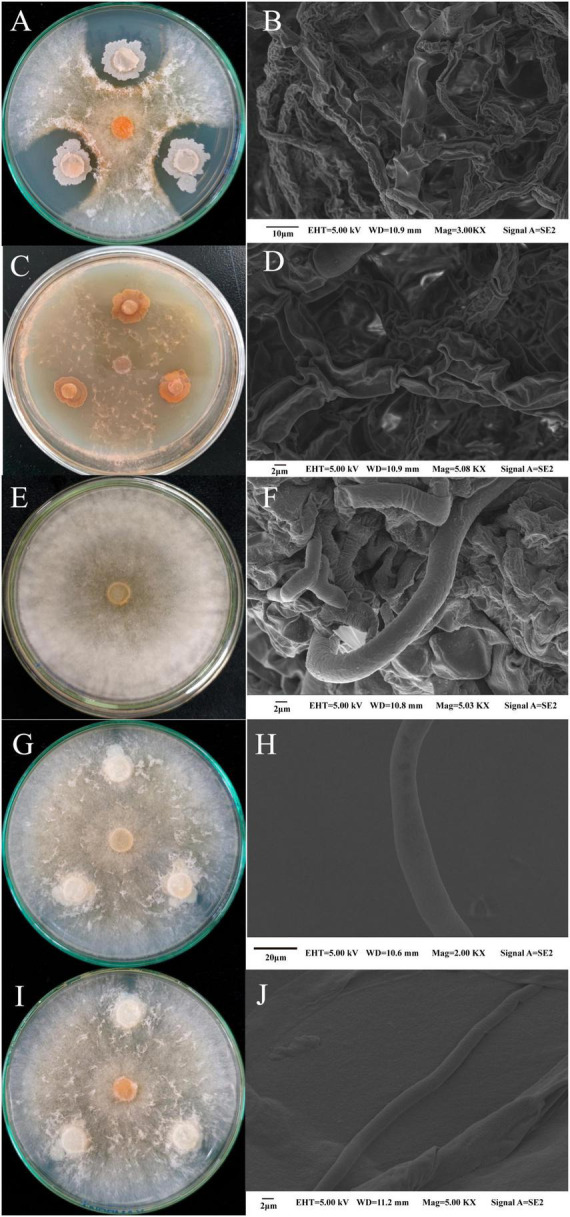
Changes in the mycelial morphology of *M. sextelata* were observed in the pathogenic bacteria and *M. sextelata* plate confrontation and through SEM: strain M-5 and *M. sextelata* confrontation **(A)** and *M. sextelata* mycelial morphology after confrontation **(B)**; strain M-B and *M. sextelata* confrontation **(C)** and *M. sextelata* mycelial morphology after confrontation **(D)**; control *M. sextelata* on the plate **(E)** and mycelial morphology observed through SEM **(F)**. Two other *B. subtilis* strains cultured in standoff with *M. sextelata* ZW111, namely *B. subtilis* B626 **(G)** and mycelial morphology observed through SEM **(H)**; *B. subtilis* B602 **(I)** and mycelial morphology observed through SEM **(J)**.

The plate standoff results (Figure 4) revealed a clear inhibition circle between the pathogenic bacteria and *M. sextelata*. Strains M-B and M-5 severely inhibited mycelial growth in *M. sextelata* (Figures 4A, C). SEM showed that the mycelium of *M. sextelata* in the control group was healthy and full (Figure 4F), whereas that at the edge of the colonies of *M. sextelata* treated with the pathogenic strains exhibited an obvious distortion, surface depression, folding, drying, and rupture (Figures 4B, D). The results thus indicated that the pathogenic bacteria changed the morphology of the *M. sextelata* mycelium. The laboratory-preserved other strains of *B. subtilis* did not produce an inhibition circle around *M. sextelata* (Figures 4G, I), the *M. sextelata* mycelium covered the growth of the *B. subtilis* cake and the mycelium was full and strong. This indicated that the other *B. subtilis* strains had no inhibitory effect on the mycelial growth of *M. sextelata* on the plate. These results preliminarily indicate that perhaps not all *B. subtilis* strains inhibit *M. sextelata* growth. Only the *B. subtilis* strain M-5 isolated from the infected *M. sextelata* exhibited an inhibitory effect on *M. sextelata*. In the following research, we will continue our in-depth investigation on whether all *B. subtilis* will inhibit the growth of *M. sextelata*. The difference between different pathogenic and non-pathogenic strains among the same species also serves as a direction for subsequent research.

### 3.5. Effect of temperature on the growth of pathogenic bacteria

**FIGURE 5:**
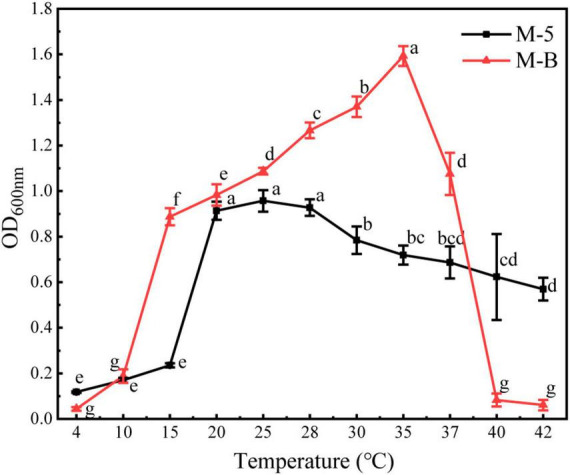
Effect of temperature on the growth of pathogenic bacteria. Different letters above values indicate significant differences (*P* < 0.05, Tukey’s test in ANOVA).

The temperature effect results showed (Figure 5 and Supplementary Tables 1, 2) that the growth of strain M-B was inhibited at 4°C. When the temperature was increased, the OD_600*nm*_ values were the maximum at 35°C, and the growth was inhibited when the temperature was higher than 40°C. Therefore, strain M-B did not grow at temperatures below 4°C or above 40°C. When the strain was incubated for 10 min in a water bath at 55°C and coated on the LB plate, no colonies were observed for 48 h, indicating that the lethal temperature for strain M-B was 55°C. The growth of strain M-5 was inhibited at 4°C, and the OD_600*nm*_ values were the maximum at 25°C with an increase in temperature. After strain M-5 was incubated on LB plates at 110°C for 10 min, no colonies were observed for 48 h, indicating that the lethal temperature for strain M-5 was 110°C. Strain M-B started to multiply at 10–15°C, and strain M-5 started at 15–20°C. Therefore, the temperature can be controlled below 15°C to as the lower temperature could inhibit the growth of pathogenic bacteria during *M. sextelata* cultivation.

### 3.6. Identification of cellulase production by pathogenic strains and determination of enzyme activity

**FIGURE 6:**
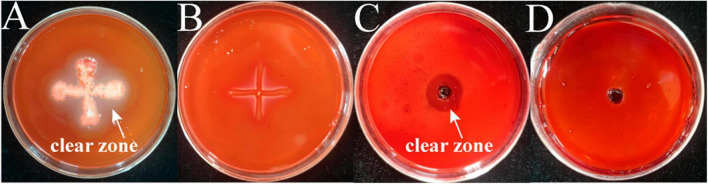
Identification of cellulase producing pathogenic strains. M-5 strain **(A)**, M-B strain **(B)**, cellulase solution positive control **(C)** and inactivated enzyme solution negative control **(D)**.

The results of cellulase secretion by two bacterial pathogens inoculated into cellulose medium by spot inoculation are shown in Figure 6. Congo red binds strongly to the large polysaccharide cellulose, which is hydrolyzed by the action of cellulase into smaller sugars that Congo red cannot bind, resulting in a hyaline ring around the colony. A clear circle can be seen around the edge of the colony of strain M-5, while no clear circle is evident around strain M-B, indicating that strain M-5 can secrete cellulase. Based on the enzyme activity data (Table 2 and Supplementary Table 5), the enzyme activity value of strain M-5 is much higher than that of strain M-B, indicating strong cellulase production ability. Strain M-5 has lower enzyme activity and weaker cellulase production ability.

**TABLE 2:** Cellulase and chitinase activity of pathogenic strains.

Strain	Cellulase activity (U/mL)	Chitinase activity (U/mL)
M-5	7.50 ± 0.29	10.62 ± 0.48
M-B	2.28 ± 0.17	11.70 ± 0.17
M-5 inactivation	0.64 ± 0.03	0.11 ± 0.07
M-B inactivation	0.50 ± 0.05	0.27 ± 0.10

Values in the table are mean ± standard deviation (*n* = 3).

### 3.7. Identification of chitinase production by pathogenic strains and determination of enzyme activity

**FIGURE 7:**
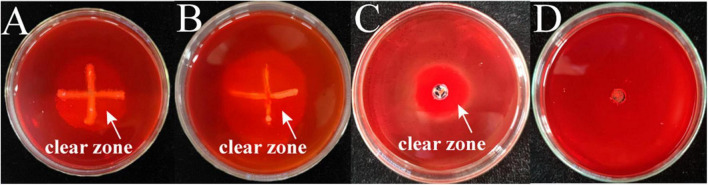
Identification of chitinase producing pathogenic strains. M-5 strain **(A)**, M-B strain **(B)**, chitinase solution positive control **(C)** and inactivated enzyme solution negative control **(D)**.

The two bacterial pathogens were inoculated separately into the chitinase medium by spot inoculation and the results of chitinase secretion by both pathogens are shown in Figure 7, where clear circles can be seen at the edge of the colonies of both strains M-5 and M-B. This indicates that both strains can secrete chitinase. Under the action of chitinase, chitin can be hydrolyzed from white insoluble particles to soluble small molecules, resulting in a transparent ring around the colony of the bacterium to be tested. Combined with the enzyme activity data (Table 2 and Supplementary Table 6), both strain M-5 and strain M-B had strong chitinase production ability.

## 4. Discussion

In recent years, *Bacillus*. spp and *Pseudomonas*. spp have been widely used as antagonists of pathogenic fungi (Haas and Défago, 2005; Lastochkina et al., 2019). In the present study, *B. subtilis* M-5 and *P. chlororaphis* subsp. *aureofaciens* M-B were identified as bacterial pathogens causing red-stipe disease of *M. sextelata*, a very unusual observation. However, in plate confrontation tests of other strains of *B. subtilis* with *M. sextelata* (Figures 4G, I), we found that not all *B. subtilis* strains inhibit *M. sextelata* growth. Only *B. subtilis* M-5, isolated from the fruiting bodies of infected morels, exerted an inhibitory effect on *M. sextelata* growth. *P. chlororaphis* subsp. *aureofaciens* exhibits inhibitory effects on fungi (Raio et al., 2011; Zhang et al., 2021). The inoculation of *M. sextelata* with *P. chlororaphis* subsp. *aureofaciens* possibly led to the inhibition of *M. sextelata* growth. Moreover, the occurrence of *M. sextelata* red-stipe disease may be related to the strong antifungal activity of *P. chlororaphis* subsp. *aureofaciens*.

*Bacillus subtilis* inhibits fungi mainly by producing numerous metabolites (Wang Z. et al., 2014; Kilani-Feki et al., 2016) including (i) bacteriocins such as chymotrypsin, polymyxin, and mycotoxin; (ii) proteases, amylases, lipases, chitinases, and other cell wall-degrading enzymes (Frändberg and Schnürer, 1994; Sadfi et al., 2001; Islam et al., 2012), which are among the more active enzymes (Islam et al., 2012; Zhang et al., 2014); and (iii) antibacterial substances such as small-molecule antimicrobial peptides, antimicrobial proteins, and polysaccharides (Xing et al., 2015). Chitin and β-l,3-glucan are enzymes crucial for fungal control, and *B. subtilis* produces chitinase and β-l,3-glucanase that degrade fungal cell walls and increase cell membrane permeability. Certain pathogenic bacteria have been shown to inhibit the growth of mycelium of morel mushrooms through antagonistic secretions (Tan et al., 2021b). In this experiment, we found that the isolated pathogenic strain M-5 could produce chitinase and cellulase, and had good enzyme-producing ability (Table 2). Antifungal proteins have a crucial role in the antagonistic effect of *B. subtilis*. These proteins can inhibit mycotoxin production, disrupt cell walls, deform the mycelium, cause abnormal spore germination, and inhibit the growth and reproduction of fungi (Rismondo and Schulz, 2021; Liu et al., 2022). A study investigating of the mechanism of inhibition of *Colletotrichum gloeosporioides* by antifungal proteins revealed that the mycelium structure after antifungal protein treatment was damaged, the cell wall was ruptured, the cytoplasmic structure was disturbed, and the contents of the cell flowed out (Liu et al., 2020; Oliveira Silva et al., 2022). Thus, antifungal proteins exert their antifungal effects by disrupting the cell wall structure and function. They also reduce the ergosterol content and affect the membrane structure and stability (Chen et al., 2023). In agreement with the present study results (Figure 4B), SEM observation of the *M. sextelata* mycelium after standoff culture with strain M-5 revealed that the mycelium structure was damaged, exhibiting dryness, wrinkling, and spillage of cell contents. This indicated that strain M-5 disrupted the cell wall of the *M. sextelata* mycelium. When the mycelial membrane integrity is disrupted, nucleic acids and proteins are released, leading to impaired cell function and loss of viability (Chen et al., 2023).

*Pseudomonas chlororaphis* is a common soil and plant root environment host bacterium that inhibits a wide range of pathogenic fungi (Raio and Puopolo, 2021). Production of several metabolites is essential for the inhibition of fungi by *P. chlororaphis*. (i) Antibiotics and antimicrobial compounds such as 2,4 diacetylphloroglucinol, hydrogen cyanide, pyrrolnitrin, pyoluteorin, and phenazines are among the most studied secondary metabolites produced by *P. chlororaphis* and many other *Pseudomonas* spp. (Arseneault and Filion, 2016; Raio and Puopolo, 2021). Phenazines have broad-spectrum antibacterial activity. These compounds are excreted as strongly diffused yellow-orange pigments during bacterial growth *in vitro* (Mavrodi et al., 2006). (ii) *P. chlororaphis* can also release volatile organic compounds (VOCs) that induce systemic resistance in plants (Ma et al., 2016). (iii) *P. chlororaphis* can produce siderophores, proteases, and lipases (Jain and Pandey, 2016). These hydrolases play a key role in the degradation of fungal cell walls and indirectly promote plant growth (Chin-A-Woeng et al., 2001). In this experiment, we found that the isolated pathogenic bacteria M-B had a strong ability to produce chitinase and a weak ability to produce cellulose (Table 2). VOCs produced by *P. chlororaphis* subsp. *aureofaciens* strain SPS-41 isolated from sweet potato roots exhibited strong antifungal activity and effectively inhibited mycelial growth and spore germination of *Ceratocystis fimbriata*, thereby causing massive cytoplasmic leakage, severely disrupting the cell membrane structure, and reducing the ergosterol content (Zhang et al., 2019, 2021). Consistent with the study results, SEM observation of the mycelial morphology of *M. sextelata* after confrontation with strain M-B (Figure 4D) revealed numerous cavities and dryness in the mycelium, thereby indicating loss of cell membrane integrity and release of cell contents. *P. chlororaphis* subsp. *aureofaciens* strain M71, isolated from tomato inter-rhizosphere, exhibited a strong inhibitory effect on *Seiridium cardinale*. Strain M71 inhibited the radial growth of fungal colonies *in vitro* by releasing phenazine compounds into the agar medium (Raio et al., 2011). In the standoff test, strain M-B also secreted a yellow-orange pigment around the colonies, indicating phenazine production.

The occurrence of bacterial diseases of *M. sextelata* during cultivation is closely related to the influence of temperature, especially high temperature and high humidity. The pathogenic bacteria are prone to attack the stalk part of *M. sextelata* ascocarp first. The infected mushroom body often produces an unpleasant fishy smell. Then, the ascocarp or the diseased part wilts, followed by reddening of the stalk, ceasing of growth, and softening of the mushroom body (Liu et al., 2019). In combination with previous studies, temperature is highly correlated with the occurrence of *M. sextelata* disease, and in this study, we found that temperature below 15°C could inhibit the growth of pathogenic bacteria. Therefore, to avoid the occurrence of bacterial diseases as much as possible, the temperature maintained during cultivation must be as low as possible so as to inhibit the growth and reproduction of pathogenic bacteria. To eliminate some pathogenic microorganisms remaining in the soil, the soil can also be disinfected at a high temperature after cultivation.

In this study, two strains, *B. subtilis* M-5 and *P. chlororaphis* subsp. *aureofaciens* M-B, were isolated from 20 samples of *M. sextelata* with red-stipe disease. The two pathogenic bacteria were identified using phylogenetic methods. This is the first study to investigate the pathogenicity, phylogenetic and morphological analysis, and biological properties of bacterial pathogens causing the *M. sextelata* red-stipe disease. Both strains exhibited strong antifungal activity in the pathogenicity test and plate confrontation test against *M. sextelata*. Both strains M-5 and M-B could produce chitinase and cellulase. *B. subtilis* M-5 and *P. chlororaphis* subsp. *aureofaciens* M-B were used here as pathogens causing bacterial diseases in *M. sextelata*. In this study, strains M-B and M-5 did cause red-stipe disease of *M. sextelata*, but what kind of relationship they have with *M. sextelata* at lower temperatures, whether it exists in *M. sextelata* or not, and whether it affects morel mushrooms in any other ways need to be further explored. In subsequent studies, we will continue to investigate the pathogenic mechanisms of *B. subtilis* and *P. chlororaphis* subsp. *aureofaciens* in *M. sextelata* bacterial diseases. We will also explore the differences between pathogenic and non-pathogenic strains and whether any factors are regulating the switch between them. The screening of antagonistic microorganisms for the *M. sextelata* red-stipe disease will also be a subsequent research direction.

## Data availability statement

The datasets presented in this study can be found in online repositories. The names of the repository/repositories and accession number(s) can be found below: NCBI–OR053654 and OR053655.

## Author contributions

XZ, SN, and KM designed the experiments. KM and JZ performed the experiments. KM, MS, and LL analyzed the data and processed the images. KM wrote the manuscript. XZ and SN performed the review and revision of the manuscript. All authors contributed to the article and approved the submitted version.
