# Supplementary material for: Isolation and identification of pathogens of Morchella sextelata bacterial disease
Source: Front Microbiol. 2023 Nov 6;14:1231353. doi: 10.3389/fmicb.2023.1231353 (PMC10657878; doi:10.3389/fmicb.2023.1231353)
Supplement: Supplementary file 1 [file Data_Sheet_1.docx]

***Supplementary Material***

Isolation and identification of pathogens of *Morchella* *sextelata* bacterial disease

**Xuetai Zhu^1*^**^†^**, Kaili Ma^1^**^†^**, Mingyue Sun^1^, Jinming Zhang^1^, Lijuan Liu^1^, Shiquan Niu^1^**

**Correspondence:** Xuetai Zhu: zhuxuetai@nwnu.edu.cn

**Accession numbers**

The GenBank accession numbers for strains M-B and M-5 are OR053654 and OR053655, respectively.

**Morphological identification of pathogens**


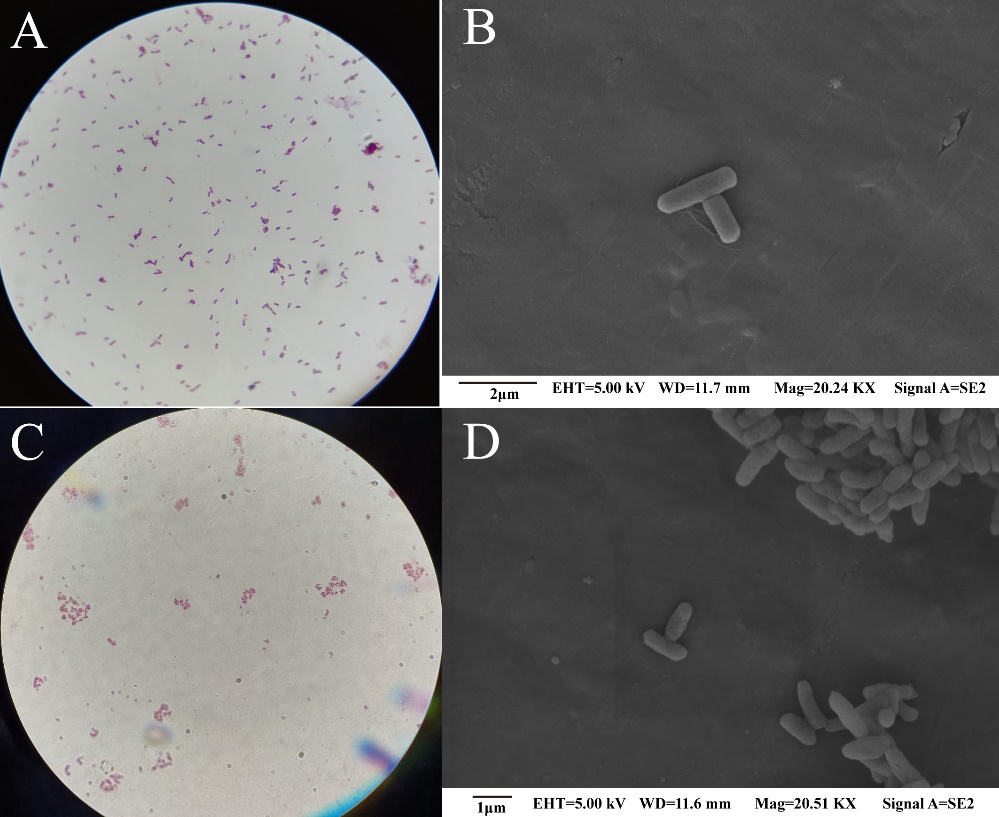


Figure 1. Gram staining results under the optical microscope (100×): M-5 **(A)** and M-B **(C).** Scanning electron microscopy results (200000×): M-5 **(B)** and M-B **(D)**.

**Table 1. Effect of temperature on the growth of strain M-5.**

| StrainM-5 | Temperature | panel data |  |  | Average value | Standard deviation | Significant difference |
| --- | --- | --- | --- | --- | --- | --- | --- |
|  | 4℃ | 0.117 | 0.114 | 0.122 | 0.118 | 0.004 | e |
|  | 10℃ | 0.179 | 0.172 | 0.158 | 0.170 | 0.011 | e |
|  | 15℃ | 0.24 | 0.225 | 0.242 | 0.236 | 0.009 | e |
|  | 20℃ | 0.926 | 0.946 | 0.869 | 0.914 | 0.040 | a |
|  | 25℃ | 0.955 | 1.006 | 0.911 | 0.957 | 0.048 | a |
|  | 28℃ | 0.912 | 0.901 | 0.969 | 0.927 | 0.037 | a |
|  | 30℃ | 0.751 | 0.749 | 0.854 | 0.785 | 0.060 | b |
|  | 35℃ | 0.767 | 0.692 | 0.698 | 0.719 | 0.042 | bc |
|  | 37℃ | 0.659 | 0.634 | 0.766 | 0.686 | 0.070 | bcd |
|  | 40℃ | 0.623 | 0.434 | 0.812 | 0.623 | 0.189 | cd |
|  | 42℃ | 0.529 | 0.625 | 0.553 | 0.569 | 0.050 | d |

**Table 2. Effect of temperature on the growth of strain M-B.**

| StrainM-B | Temperature | panel data |  |  | Average value | Standard deviation | Significant difference |
| --- | --- | --- | --- | --- | --- | --- | --- |
|  | 4℃ | 0.034 | 0.05 | 0.047 | 0.044 | 0.009 | g |
|  | 10℃ | 0.179 | 0.164 | 0.222 | 0.188 | 0.030 | g |
|  | 15℃ | 0.931 | 0.87 | 0.862 | 0.888 | 0.038 | f |
|  | 20℃ | 0.992 | 0.932 | 1.025 | 0.983 | 0.047 | e |
|  | 25℃ | 1.075 | 1.083 | 1.104 | 1.087 | 0.015 | d |
|  | 28℃ | 1.26 | 1.236 | 1.304 | 1.267 | 0.034 | c |
|  | 30℃ | 1.416 | 1.369 | 1.326 | 1.370 | 0.045 | b |
|  | 35℃ | 1.589 | 1.552 | 1.638 | 1.593 | 0.043 | a |
|  | 37℃ | 0.969 | 1.128 | 1.131 | 1.076 | 0.093 | d |
|  | 40℃ | 0.065 | 0.115 | 0.067 | 0.082 | 0.028 | g |
|  | 42℃ | 0.081 | 0.065 | 0.036 | 0.061 | 0.023 | g |

**Table 3. Glucose standard curve.**

| Standard Sugar Solution (1mg/mL) | OD540nm |  |  | Average value |
| --- | --- | --- | --- | --- |
| 0.4 | 0.23 | 0.22 | 0.25 | 0.233 |
| 0.8 | 0.464 | 0.472 | 0.466 | 0.467 |
| 1.2 | 0.68 | 0.696 | 0.693 | 0.690 |
| 1.6 | 0.897 | 0.913 | 0.906 | 0.905 |
| 2 | 1.113 | 1.118 | 1.121 | 1.117 |

**Table 4. N-acetyl-D-glucosamine standard curve.**

| Standard Sugar Solution (1mg/mL) | OD540nm |  |  | Average value |
| --- | --- | --- | --- | --- |
| 0.2 | 0.038 | 0.041 | 0.039 | 0.039 |
| 0.4 | 0.127 | 0.126 | 0.128 | 0.127 |
| 0.6 | 0.205 | 0.203 | 0.204 | 0.204 |
| 0.8 | 0.285 | 0.291 | 0.3 | 0.292 |
| 1.0 | 0.361 | 0.368 | 0.377 | 0.367 |
| 1.2 | 0.44 | 0.451 | 0.445 | 0.445 |

**Table 5. Determination of cellulase activity.**

| strain | OD540nm |  |  | enzyme activity |  |  | average value | standard deviation |
| --- | --- | --- | --- | --- | --- | --- | --- | --- |
| M-B | 0.191 | 0.212 | 0.228 | 2.099 | 2.292 | 2.439 | 2.276 | 0.171 |
| M-5 | 0.813 | 0.769 | 0.753 | 7.816 | 7.412 | 7.265 | 7.497 | 0.286 |
| M-B inactivation | 0.013 | 0.015 | 0.022 | 0.463 | 0.481 | 0.546 | 0.497 | 0.047 |
| M-5 inactivation | 0.029 | 0.035 | 0.033 | 0.610 | 0.665 | 0.647 | 0.643 | 0.031 |

**Table 6. Determination of chitinase activity.**

| strain | OD540nm |  |  | enzyme activity |  |  | average value | standard deviation |
| --- | --- | --- | --- | --- | --- | --- | --- | --- |
| M-B | 2.487 | 2.428 | 2.445 | 11.884 | 11.560 | 11.654 | 11.698 | 0.168 |
| M-5 | 0.892 | 0.838 | 0.905 | 10.801 | 10.069 | 10.977 | 10.617 | 0.482 |
| M-B inactivation | 0.115 | 0.121 | 0.107 | 0.277 | 0.359 | 0.169 | 0.270 | 0.095 |
| M-5 inactivation | 0.096 | 0.108 | 0.103 | 0.047 | 0.183 | 0.115 | 0.113 | 0.065 |
